# Trends in Treatment of Active, Moderate-to-Severe Thyroid Eye Disease in the United States

**DOI:** 10.1210/jendso/bvaa140

**Published:** 2020-09-25

**Authors:** Yao Wang, Anu Sharma, Lissa Padnick-Silver, Megan Francis-Sedlak, Robert J Holt, Colleen Foley, Guy Massry, Raymond S Douglas

**Affiliations:** 1 Cedars Sinai Medical Center, Los Angeles, California; 2 Division of Endocrinology, Diabetes and Metabolism, University of Utah School of Medicine, Salt Lake City, Utah; 3 Horizon Therapeutics plc, Lake Forest, Illinois

**Keywords:** Graves’ ophthalmopathy, disease management, referral patterns

## Abstract

**Introduction:**

Limited data exist on US referral/management patterns for moderate-to-severe thyroid eye disease (TED), a disabling condition.

**Methods:**

US ophthalmologists and endocrinologists experienced in treating TED provided medical record data of moderate-to-severe TED patients and information on referral/treatment practices. Data on signs/symptoms, medical/surgical treatments, treatment response, and referral history were collected. Moderate and severe cases were stratified to interrogate treatment/practice differences.

**Results:**

A total of 181 physicians provided data on 714 patients (49.4 ± 13.6 years old, 65% women, 14% severe disease). Reporting physicians diagnosed 55% of patients themselves and solely managed 37% of cases, with similar referral/comanagement patterns between moderate and severe cases. Topical therapies included lubricating (79%) and glucocorticoid (39%) eye drops. Systemic therapies included oral glucocorticoids (36%), IV glucocorticoids (15%), and rituximab and/or tocilizumab (12%). Few patients underwent orbital radiation (4%) or surgical intervention (4%). IV glucocorticoids (33% vs. 12%), biologics (26% vs. 10%), orbital radiation (11% vs. 3%), and ocular surgery (12% vs. 3%) were used more often in severe versus moderate cases (all *P* < 0.001). However, severe disease was less responsive to therapy (very responsive to therapy: 28% vs. 49%, *P* < 0.001).

**Conclusions:**

Participating physicians were primarily responsible for just over one-half of TED diagnoses, but solely treated <40% of patients. Severe TED was treated more often with surgery and systemic immunologic therapies than moderate disease, but was less likely to respond to treatment. These results reinforce that moderate-to-severe TED is difficult to treat with an unmet medical need in the United States.

Thyroid eye disease (TED) is a serious, debilitating, autoimmune condition that results in retro-orbital inflammation and subsequent proptosis, diplopia, corneal exposure, vision changes, and potential blindness [1, 2]. TED is listed as a rare condition by the National Organization of Rare Disorders [3] and described as a “rare disease” by the American Academy of Ophthalmology [4]. Severe disease is even more rare, accounting for only 3% to 5% of all TED cases [5]. Patients initially present with active inflammation, including ocular redness, pain around and behind the eyes, and periorbital tissue redness and swelling. Progressive inflammation often results in significant changes to retro-orbital fat and muscles resulting in proptosis and/or diplopia and, in sight-threatening cases, optic nerve compression and corneal disease.

Patient quality of life [6-10] and mental health [11-14] are drastically affected by TED, with quality of care playing a large role [15]. Unfortunately, little is known about physician referral and treatment patterns in the United States, but patients with active, progressive TED may be treated with a wide range of therapies, with interventions widely varying from lubricating eye drops to systemic glucocorticoids to emergency orbital decompression surgery.

European guidelines recommend managing mild TED with local measures, selenium supplementation, and a “watch and wait” approach [16]. For active, moderate-to-severe disease, systemic glucocorticoids are used as a first-line therapy in Europe, preferably IV [16]. In contrast, the American Thyroid Association 2016 guidelines focus on treating thyroid issues and do not make specific recommendations for treating TED [17]. Because of the absence of US TED-specific treatment guidelines, limitations of off-label therapies, and a previous lack of US Food and Drug Administration-approved treatments, the usual practice patterns surrounding TED are not well understood in the United States [5]. Further, both endocrinologists and ophthalmologists manage patients with TED, but little is known about referral/management patterns. Here, we report the results of the first survey on TED patient management and treatment trends completed in the United States involving a large number of physicians who routinely treat TED.

## Methods

Ophthalmologists and endocrinologists across the United States were asked to provide retrospectively collected chart data on up to 4 patients with moderate-to-severe TED (Table 1). Stepwise patient filtration was used to identify potential duplicate patients, including the state in which the reporting physician practiced in, patient sex, patient race, patient age, TED duration, smoking status, and diabetes status. Patient and physician data were collected in late 2018, before the US Food and Drug Administration approval of teprotumumab. Though patients with mild TED were not included in the retrospective medical record review, participating physicians provided information on their usual referral practices regarding TED cases of all severities. All participating physicians were experienced with TED, having directly managed this rare disorder in at least 5 cases in the 12 months before data collection.

**Table 1.** Applicable Survey Items

	Question	Response Options
*Physician-reported items*		
	How much of your professional time do you spend in each of the following settings?	Office-based practice, academic/teaching hospital, community hospital (for profit), community hospital (not for profit), VA/government hospital, other
	Which of the following best describes your office-based practice?	Solo private practice, group private practice (single specialty), group private practice (multi-specialty), office-based practice affiliated with an academic hospital, office-based practice affiliated with a community hospital, other
	Thinking of your mild, moderate, and severe, active TED patients, what percentage fall into the following categories?	I personally treat and do not refer out or consult with any other specialists on TED treatment.
		Consult with other specialist(s) and comanage the patient for TED treatment.
		Refer the patient out completely to another specialist(s) for TED treatment.
*Retrospective chart review items*		
	What is the current disease severity?	Mild, moderate, severe
	What was the specialty of the physician who officially diagnosed TED in this patient?	I personally diagnosed this patient.
		(Another) ophthalmologist diagnosed this patient.
		(Another) endocrinologist diagnosed this patient.
		(Another) ocular surgeon diagnosed this patient.
		Other
	What type of physician referred this patient to you, if at all?	PCP/IM, optometrist, endocrinologist, ophthalmologist, other, self-referral by patient,
	What type of physician did you refer this patient out to, if at all?	PCP/IM, optometrist, endocrinologist, ophthalmologist, other, patient not referred out
	Which of the following does the patient experience currently? (check yes or no)	Eye pain in primary gaze, eye pain with ocular movement, conjunctival redness/injection, light sensitivity/photophobia, eye dryness, gritty sensation, tearing, blurred vision, diplopia/double vision, color vision changes, vision loss, decrease in visual acuity since baseline, soft-tissue involvement, exophthalmos/proptosis, strabismus/misalignment of eye, eyelid erythema/redness, eyelid swelling, chemosis/swelling of conjunctiva, eyelid retraction from baseline, corneal involvement, periorbital edema/swelling around the eye, compressive optic neuropathy, eye muscle involvement
	In the grid below, please indicate the patient’s current and previous treatment regimens for TED. (information for 2 prior treatment regimens requested)	Non-Rx treatment (OTC therapies [eg, artificial tears]), prescription lubricating eye drops, steroid eye drops, oral steroids, IV steroids, periorbital/peribulbar steroid injection, rituximab (Rituxan), tocilizumab (Actemra), orbital radiation, ocular surgeries/procedures, other therapy, no Rx or non-Rx treatment.
	How would you characterize the patient’s response to the patient’s **current regimen**?	Responding really well
		Responding somewhat
		Responding poorly

**Abbreviations: **D Non-Rx, non-prescription; OTC, over the counter; PCP/IM, primary care/internal medicine physician; Rx, prescription; TED, thyroid eye disease; VA, veterans affairs.

TED activity and TED severity were assessed as separate disease measures. Activity was determined by calculating the clinical activity score (CAS), where 1 point was given for each of the following TED signs/symptoms present: eyelid redness, eyelid swelling, conjunctival redness, conjunctival swelling, pain in the primary gaze, pain with eye movement, and caruncle swelling [17]. In the current study, 6 of 7 CAS measures were used because caruncle status was not reported. Physicians rated TED severity as moderate or severe based on clinical assessment (specific severity rating instructions were not given) and were asked to list current treatments (from a list), current treatment regimen response, and previous treatment regimens (up to 2 prior treatment regimens could be included if applicable; Table 1). Patient treatment response was evaluated by answering, “How would you characterize the patient’s response to the patient’s current regimen?” Physicians answered “responding really well,” “responding somewhat,” or “responding poorly.”

The following demographic data were collected and assessed for the prevalence of severe versus moderate TED: gender, age, duration of TED, and smoking status. Data are presented as mean ± standard deviation and n (%) as appropriate. Differences between physician responses and clinical characteristics were compared between groups using Student *t* tests for continuous variables and Fisher’s exact tests for categorical variables. Statistical significance was defined as *P* < 0.05.

This study was reviewed by the Western Institutional Review Board (Puyallup, WA) and was assigned exempt status, waiving the requirement of informed consent. All study conduct adhered to the tenets of the Declaration of Helsinki.

## Results

### Physician and patient populations

A total of 181 physicians (73 endocrinologists, 108 ophthalmologists) participated in the study, providing deidentified data on 714 patients. Only 4 potential duplicate pairs (0.6% of study population) were initially identified with stepwise filtration, all of which were assessed to have different treatment histories and, therefore, were unlikely duplications. Ophthalmology subspecialists included oculoplastic surgeons (n = 44), corneal specialists (n = 14), neuro-ophthalmologists (n = 6), and strabismus specialists (n = 3). The remaining ophthalmologists were general ophthalmologists (n = 36) or listed “other” as their subspecialty (n = 5). All physicians were board-certified, experienced practitioners (15.0 ± 7.0 years postresidency [range: 4-30 years]), and highly experienced in managing TED (minimum of 10 TED cases reported within 12 months before data collection [of any activity or severity]). Most physicians practiced within a single (36%) or multispecialty (44%) group and spent the majority of their professional time (83%) in an office-based setting. A small proportion of physicians (6%) reported that their practices were affiliated with an academic hospital. Participating physicians were geographically well distributed, practicing in 39 states.

All included patients were diagnosed with active, moderate-to-severe TED, with an average TED duration of 4.2 ± 5.1 years and severe disease noted in 102 patients (14%). Moderate and severe TED patients had similar characteristics, but significantly more patients with severe TED were current smokers (21% vs. 11%, *P* = 0.009; Table 2). The majority of included patients were euthyroid at data collection (555 patients [78%]) and had been diagnosed with Graves’ disease (475 patients [67%], Graves’ duration: 6.4 ± 9.1 years), Hashimoto thyroiditis (13 patients [2%]), or both (12 patients [2%]). There were no characteristic differences between euthyroid and noneuthyroid patients, but patients with Graves’ disease were younger at data collection (48.3 ± 13.3 vs. 51.8 ± 13.7 years, *P* = 0.002) and TED diagnosis (44.3 ± 12.6 vs. 47.3 ± 13.1 years, *P* = 0.004) and had severe TED more often (17% vs. 9%, *P* = 0.004) than patients without a Graves’ disease diagnosis.

**Table 2.** Characteristics of Patients With Thyroid Eye Disease Who Had Been Diagnosed With Active, Moderate-to-Severe Disease

	All Patients (n = 714)	Moderate TED (n = 612)	Severe TED (n = 102)	*P* ^*a*^
% patients	—	86%	14%	–
Women	466 (65%)	398 (65%)	68 (67%)	.823
Age, y	49.4 ± 13.6	49.5 ± 13.7	49.4 ± 12.7	.949
TED duration, y	4.2 ± 5.1	4.2 ± 5.1	4.2 ± 5.2	.979
Smoking status^*b*^				
Never smoked	341 (48%)	292 (48%)	49 (48%)	>.999
Former smoker	237 (33%)	211 (35%)	26 (26%)	.088
Current smoker	88 (12%)	67 (11%)	21 (21%)	.009

Data are presented as mean ± standard deviation and n (%) as applicable.

^*a*^Comparison between moderate and severe patients.

^*b*^The reporting physician was unsure of smoking status in 42 moderate and 6 severe patients.

TED, thyroid eye disease.

### Physician diagnosis and management trends

**Figure 1.**
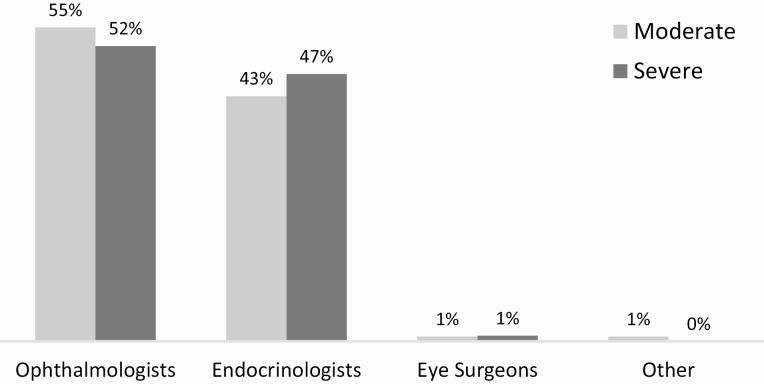
Physicians’ perspective on who is diagnosing moderate and severe active thyroid eye disease (TED). Reporting oculoplastic surgeon diagnoses accounted for 12% of ophthalmologist diagnoses.

Patients had been diagnosed with TED more often by an ophthalmologist than by an endocrinologist (55% vs. 44%), with the remaining cases diagnosed by a nonparticipating ocular surgeon (1%) or other type of physician (1%). When cases were examined by TED severity, no significant differences in diagnosis patterns emerged between moderate and severe patients (Fig. 1). Patients were most often referred to the participating doctor by a primary care physician (44%), with equal referral by an ophthalmologist (18%) or endocrinologist (18%). A small proportion of patients were self-referrals (12%) or had been referred by an optometrist (8%). There were no significant differences in referral patterns between moderate and severe TED patients.

**Figure 2.**
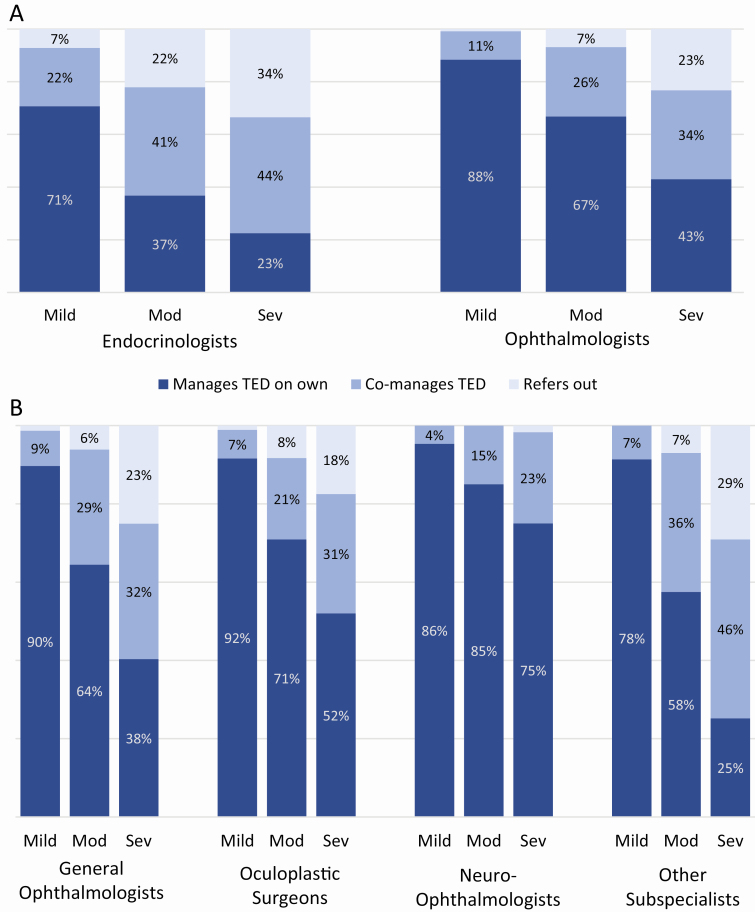
Physician-reported referral/management trends for treating/managing active thyroid eye disease. (A) Trends among endocrinologists (n = 73) and ophthalmologists (n = 108) are shown. (B) Trends among ophthalmologists most likely to manage and/or treat thyroid eye disease (general ophthalmologists [n = 36], oculoplastic surgeons [n = 44], and neuro-ophthalmologists [n = 6]), including other ophthalmology subspecialists (strabismus, cornea, and “other” subspecialists; n = 22), are also shown. Mod, moderate; Sev, severe; TED, thyroid eye disease.

Referrals by participating physicians to other providers for managing/treating TED increased as TED severity increased. This finding represents only the ophthalmic/orbital management and not thyroid-related referrals (because the latter were not captured). When asked about overall treatment/referral patterns, physicians reported that they solely manage/treat the majority of mild active TED patients (81%). However, they indicated that they solely manage about one-half (55%) of moderate and one-third (35%) of severe TED patients. A similar trend was observed for endocrinologists and ophthalmologists, but ophthalmologists were more likely to be part of patient care (managed TED on own/co-managed TED) for all TED severities (Fig. 2A). Physician-reported management trends were similar among general ophthalmologists, oculoplastic surgeons, neuro-ophthalmologists, and other ophthalmology subspecialists (strabismus surgeons, corneal specialists, and “other” subspecialists; Fig. 2B).

### Treatment of active thyroid eye disease

#### Current treatments.

At the time of data collection, the vast majority of patients (85%) were being treated with topical ophthalmic therapies, including nonprescription lubricating (55%), prescription lubricating (54%), and glucocorticoid (39%) eye drops. Additionally, 51% of patients were being treated with a systemic therapy (oral glucocorticoids: 36%, IV glucocorticoids: 15%, rituximab/tocilizumab: 12%) and 12% of patients were being treated with an orbital therapy (periorbital glucocorticoids: 6%, orbital radiation: 4%, ocular surgery: 4%).

**Figure 3.**
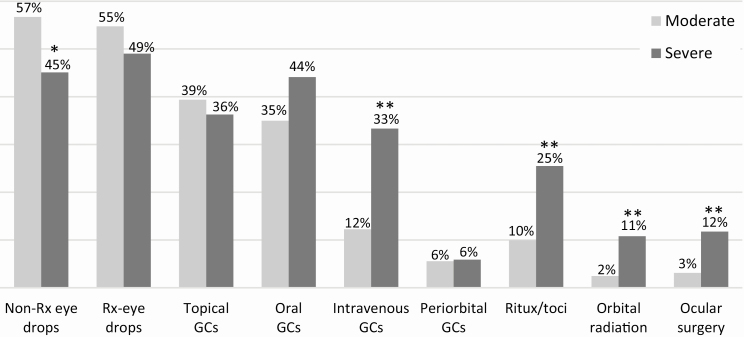
Treatments used by US ophthalmologists and endocrinologists to treat patients with active, moderate-to-severe thyroid eye disease (714 patients). Treatments shown represent therapies currently being used at the time of data collection (prior therapies not included). Significant differences between moderate and severe patients noted by *P = 0.03 and **P < 0.001. GC, glucocorticoids; non-Rx, nonprescription lubricating; Ritux/toci, rituximab and/or tocilizumab; Rx, prescription lubricating.

A larger percentage of patients with severe TED than moderate TED were currently being treated with the following therapies: IV glucocorticoids (33% vs. 12%), rituximab and/or tocilizumab (26% vs. 10%), orbital radiation (11% vs. 3%), and ocular surgery (12% vs. 3%, all *P* < 0.001; Fig. 3). Prescription lubricating and glucocorticoid eye drops, oral glucocorticoids, and periorbital glucocorticoids were similarly used to treat moderate and severe TED cases. Even though severe TED was treated more aggressively, physicians perceived only 28% of severe TED patients to have responded “really well” to therapy compared with 49% of moderate TED patients.

#### Use of systemic glucocorticoids.

**Figure 4.**
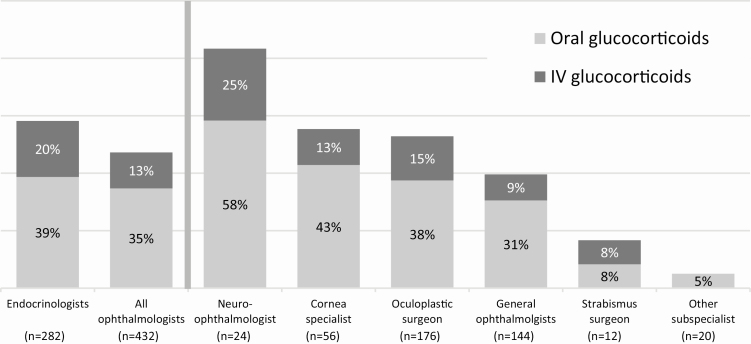
The proportion of patients currently (at the time of data collection) being treated with oral and/or IV glucocorticoids by specialty and subspecialty. “n” represents the number of patients reported by each specialty/subspecialty. “All ophthalmologists” includes general and ophthalmology subspecialty patients.

Patients were stratified by current systemic (oral and/or IV) glucocorticoid use to better understand what disease signs and symptoms may be driving the use of systemic glucocorticoids. (Table 3). A total of 329 patients (46%) were currently being treated with systemic glucocorticoids at the time of data collection (oral: 259 patients [36%], IV: 109 patients [15%]). Patients of endocrinologists, neuro-ophthalmologists, corneal specialists, and oculoplastic surgeons were most likely to currently be treated with systemic glucocorticoids (Fig. 4).

**Table 3.** Characteristics of Patients With and Without Concomitant Glucocorticoid Use (Patients Were Classified Using Current Treatment Regimen)

	No Current Systemic Glucocorticoid Use (n = 385)	Currently Treated with Oral Glucocorticoids (n = 259)	Currently Treated with IV Glucocorticoids (n = 109)
% all patients (714 patients)	54%	36%	15%
Women	265 (69%)	159 (61%)	64 (59%)
Age, y	49.9 ± 13.6	48.4 ± 13.3	50.6 ± 14.6
TED duration, y	4.8 ± 5.8	3.5 ± 4.1	3.4 ± 4.1
Severe disease	37 (10%)	45 (17%)	34 (31%)
TED activity			
CAS	1.9 ± 1.7	2.6 ± 1.9	3.4 ± 2.0
CAS ≥ 3	131 (34%)	130 (50%)	74 (68%)
Pain symptoms			
Pain in primary gaze (CAS)	68 (18%)	67 (26%)	49 (45%)
Pain with eye movement (CAS)	89 (23%)	97 (38%)	57 (52%)
Photophobia	125 (33%)	113 (44%)	57 (52%)
Vision disturbances			
Blurred/decreased vision	141 (37%)	116 (45%)	66 (61%)
Diplopia	87 (23%)	74 (29%)	53 (49%)
Color vision changes	37 (10%)	40 (15%)	28 (26%)
Structural issues			
Soft-tissue swelling^*a*^	229 (60%)	194 (75%)	93 (85%)
Proptosis	250 (65%)	182 (70%)	76 (70%)
Strabismus	77 (20%)	74 (29%)	48 (44%)
Eyelid redness (CAS)	135 (35%)	122 (47%)	62 (57%)
Eyelid swelling (CAS)	131 (34%)	115 (44%)	64 (59%)
Conjunctival swelling (CAS)	113 (29%)	110 (43%)	62 (57%)
Conjunctival redness (CAS)	213 (55%)	170 (66%)	79 (73%)
Eyelid retraction	94 (24%)	88 (34%)	46 (42%)
Corneal involvement	98 (26%)	83 (32%)	49 (45%)
Compressive optic neuropathy	25 (7%)	29 (11%)	26 (24%)
Eye muscle involvement	111 (29%)	96 (37%)	58 (53%)
Ever treated with (current or prior use)			
Intravenous GCs	82 (21%)	71 (27%)	109 (100%)
Oral GCs	144 (37%)	259 (100%)	71 (65%)
Periorbital GCs	42 (11%)	37 (14%)	27 (25%)
Topical GCs (eye drops)	220 (57%)	149 (58%)	64 (59%)
Rituximab and/or tocilizumab	51 (13%)	36 (14%)	34 (31%)
Orbital radiation	29 (8%)	25 (10%)	19 (17%)
Ocular surgery	51 (13%)	18 (7%)	14 (13%)

Data presented as mean ± standard deviation or n (%) as appropriate. Thirty-nine patients were currently being treated with both oral and IV glucocorticoids and are included in both GC groups.

^***a***^Includes soft-tissue involvement, eyelid swelling, and/or periorbital swelling.

CAS, clinical activity score; GC, glucocorticoid; TED, thyroid eye disease.

More patients who were currently being treated with systemic glucocorticoids at the time of survey had pain (primary gaze: 30% vs. 18%, with eye movement: 40% vs. 23%, photophobia: 45% vs. 33%; all *P* ≤ 0.001), visual disturbances (decreased vision: 48% vs. 37%, diplopia: 33% vs. 23%; both *P* = 0.002), and soft-tissue swelling (77% vs. 60%, *P* < 0.001) than those who were not. Both proptosis (71% vs. 65%, *P* = 0.092) and a dry/gritty sensation (84% vs. 86%, *P* = 0.978) were highly prevalent in both groups.

**Figure 5.**
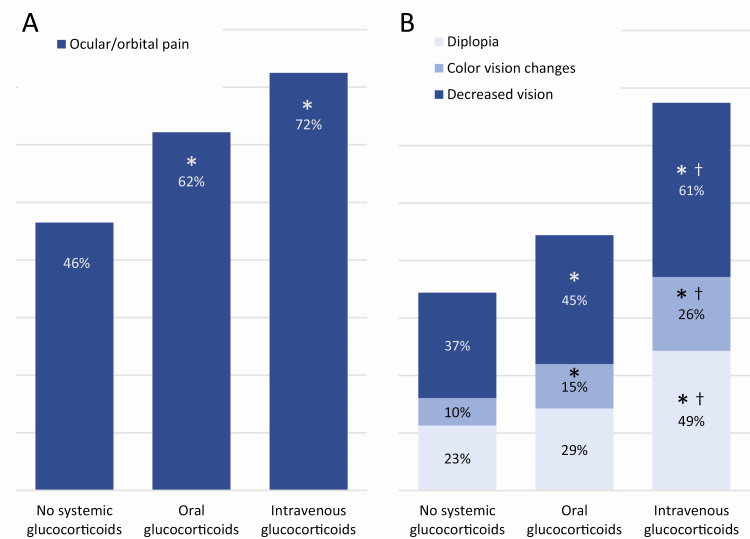
Proportion of patients with reported (A) pain and (B) visual symptoms in those who were and were not currently being treated with systemic glucocorticoids. *Indicates a significant difference from patients not treated with a systemic glucocorticoid (*P* ≤ 0.04). ^†^Indicates a significant difference from patients currently being treated with oral glucocorticoids (*P* ≤ 0.027). Ocular/orbital pain represents pain in the primary gaze, pain with eye movement, and/or photophobia. Decreased vision represents a decrease in visual acuity, blurry vision, and/or vision loss.

Further, patients being treated with IV glucocorticoids had a higher CAS (3.4 ± 2.0) than those receiving oral glucocorticoids (2.6 ± 1.9, *P* < 0.001) and those not currently being treated with a systemic glucocorticoid (1.9 ± 1.7, *P* < 0.001). Additionally, patients treated with IV glucocorticoids had more pain and visual disturbances than patients treated with oral glucocorticoids and those not treated with systemic glucocorticoids. Patients treated with IV glucocorticoids had orbital/ocular pain (pain in primary gaze, pain with eye movement, and/or photophobia; 72% vs. 46%), decreased vision (61% vs. 37%), diplopia (49% vs. 23%), and color vision changes (26% vs. 10%) more often than those who were not being treated with systemic glucocorticoids (all *P* < 0.001; Fig. 5). Exposure keratopathy symptoms (ocular dryness/grittiness [83%-86%], excessive tearing [57%-66%]) were heavily present in all groups.

Of the 109 patients on IV glucocorticoids, 27 (25%) were concomitantly receiving some sort of orbital therapy (retro-orbital glucocorticoids, orbital radiation, and/or ocular surgery) and 39 (36%) were concomitantly receiving oral glucocorticoids. Additionally, 71 patients (65%) had received oral glucocorticoids at some point during TED treatment. However, physicians reported that only 31% of these patients were responding “really well” to therapy. In contrast, only 27% of patients currently being treated with oral glucocorticoids had received a course of IV glucocorticoids previously.

#### Current and former treatments.

**Figure 6.**
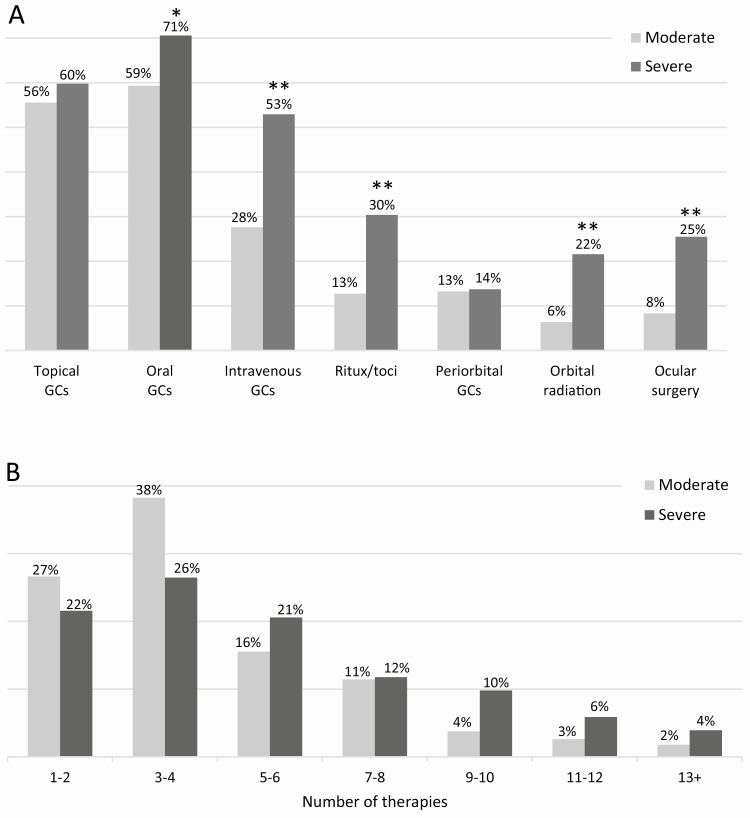
(A) Current and former treatments used on patients with active, moderate-to-severe thyroid eye disease, as reported by physicians in the United States. Treatments shown here represent both current and former therapies used. Significant differences between moderate and severe patients noted by *P = 0.037 and **P < 0.001. GC, glucocorticoid; Ritux/toci, rituximab and/or tocilizumab. (B) Total number of current and former therapies used to treat thyroid eye disease signs and symptoms. The median number of treatments was 3 and 5 for patients with moderate and severe thyroid eye disease, respectively, indicating a difference in the 2 distributions. Note: The same therapy may have been counted multiple times if listed under both current and former treatments.

Topical lubricating and/or glucocorticoid therapies had been used in the majority of patients at some point during treatment of TED (86% and 56%, respectively). Orbital therapies, (periorbital glucocorticoids, orbital radiation, and orbital surgery) were not used often (treatment rate of 9%-13%), but systemic glucocorticoids had been used at some point in 71% of patients. Nearly twice as many patients were treated with oral glucocorticoids (61%) than IV glucocorticoids (31%). Additionally, systemic glucocorticoid use was higher in severe patients than in moderate ones (oral: 71% vs. 59%, *P* = 0.037, IV: 53% vs. 28%, *P* < 0.001; Fig. 6A). Rituximab and/or tocilizumab (30% vs. 13%) and orbital radiation (22% vs. 6%) had also been used more often in severe patients than in moderate ones at some point during treatment (both *P* < 0.001).

#### Number of therapies administered.

A large number of current and former therapies were used to manage TED signs and symptoms. Physicians had used an average of 4.3 ± 2.9 therapies (median = 3, range: 1-17) on moderate TED patients and 5.4 ± 3.5 therapies (median = 5, range: 1-15) on severe TED patients (Fig. 6B). Furthermore, 7 or more therapies had been used by nearly one-third (31%) of severe and one-fifth (20%) of moderate cases and 10 or more therapies had been used in 17% of severe patients. It should be noted that the same therapy was counted more than once if reported multiple times in the same patient.

## Discussion

There is a paucity of treatment and referral data on US patients with moderate-to-severe thyroid eye disease. Here, we examined diagnosis, referral, and treatment patterns of 714 patients reported by 181 US physicians. Ophthalmologists diagnosed slightly more than one-half of patients, with the remaining patients largely diagnosed by endocrinologists. These diagnosis trends did not differ between moderate and severe cases of TED. Additionally, 99% of included patients were diagnosed by an endocrinologist or ophthalmologist, with 44% of patients referred by a primary care physician. This finding indicates that primary care providers referred most patients to the reporting physician without a formal TED diagnosis. Referral rates (either for complete referral or for comanagement with another physician) increased as TED severity increased, presumably because of the complexity and specific treatment needs of patients with severe disease. The most frequently used therapeutic category was topical ophthalmic drops. Most patients were currently being treated with some form of topical therapy. These included over-the-counter and/or prescription ocular surface lubricating therapies, likely to manage symptoms associated with dry eye and exposure keratopathy, which were highly prevalent. Topical glucocorticoids were currently being used in more than one-third of patients to help manage active inflammation, including eye dryness and redness, which was present in nearly two-thirds of patients.

More than one-half of the patients had previously been on glucocorticoid therapy, indicating suboptimal relief of symptoms. Pain and visual symptoms appear to be driving the utilization of systemic glucocorticoids rather than classic inflammatory symptoms. If true, this is surprising because there are no reliable data on glucocorticoids reducing progressive TED sequelae (eg, proptosis [18-20], strabismus/diplopia [18]). The use of oral (36%) and IV (15%) glucocorticoids is different in the United States than in Europe, where IV glucocorticoids are almost universally used to treat active, moderate-to-severe TED and are heavily favored/recommended over oral glucocorticoids [16]. This treatment practice has not been widely advocated in the United States and providers most often choose to treat active, moderate-to-severe TED predominately with topical or oral glucocorticoids, which are more readily available, convenient, and tolerable for the patient as compared with IV glucocorticoids. Our data indicate that IV glucocorticoids are reserved in the United States mostly for the severest of cases or when oral glucocorticoids are not effective. Patients of endocrinologists were treated with oral and IV glucocorticoids slightly more often than patients of ophthalmologists. However, there was large variation in glucocorticoid use within the ophthalmology community, with patients of neuro-ophthalmologists treated most often with IV and oral glucocorticoids.

More aggressive therapies, including rituximab and/or tocilizumab, orbital radiation, and ocular surgery (data not shown), were used more often by ophthalmologists than by endocrinologists. Additionally, patients with severe TED were currently being treated with systemic treatments and aggressive ocular therapies more often than patients with moderate TED, including IV glucocorticoids, rituximab and/or tocilizumab, orbital radiation, and ocular surgery. Differences in the proportion of smokers between moderate and severe TED groups may have contributed. In contrast, topical therapies and oral glucocorticoids were used similarly in both moderate and severe patients. Our analyses also revealed that physicians are using a large number of therapies to treat TED, particularly in severe cases. Severe TED patients were treated with a median of 5 therapies and approximately one-third of cases had been treated with 7 or more therapies. Despite more aggressive therapy, physicians reported that only about one-quarter of severe TED patients responded “really well” to treatment, which was significantly lower than in moderate TED patients. These findings are indicative of the unmet need at the time of survey for treatments that improve TED-related inflammation and its orbital sequelae. Teprotumumab has been shown to improve inflammation, proptosis, and diplopia associated with TED [21, 22] and its recent approval may change the TED treatment paradigm. Therefore, a repeat of the current study may be needed in the near future.

This study had several strengths. First, it included a large number of experienced physicians who regularly treat TED and a large number of their patients with a current diagnosis of moderate-to-severe TED. To the best of our knowledge, this is the first such analysis of this population in the United States. Additionally, our study cohort was characteristic of the greater TED population, as indicated by matching demographic characteristics. This study had several limitations, mostly relating to its survey nature and retrospective design, though physicians were reporting on currently managed patients. Additionally, diagnosis trends, referral patterns, TED severity, and treatment responses may have been influenced by physician opinion and/or patient record accuracy.

In conclusion, this study provides insight into how a large number of patients with active, moderate-to-severe TED were diagnosed, managed, and treated in the United States. Treatment approaches in late 2018 were highly variable, likely because of a lack of effective treatment options and the absence of clear TED management/treatment guidelines for physicians in the United States. Further multicenter studies are needed to verify these findings and to provide further insight into how physicians are managing and treating this rare, difficult-to-treat condition in the United States. Finally, these results indicate that moderate-to-severe TED represents a significant unmet medical need.

## Data Availability

The datasets generated during and/or analyzed during the current study are not publicly available, but are available from the corresponding author on reasonable request.

## Abbreviations

CAS: clinical activity score
TED: thyroid eye disease

## Additional Information

***Disclosure Summary:*** L.P.-S., M.F.-S., R.J.H., and C.F. employees of, and have stock in, Horizon Therapeutics plc. R.S.D. is a consultant for Horizon Therapeutics. The remaining authors have no conflicts of interest to disclose.

***Meeting presentations:*** Preliminary data were presented in a poster at the 2019 Annual Meeting of the American Thyroid Association (November 1, 2019; Chicago, IL).
